# Cardiorenal Disease in COVID-19 Patients

**DOI:** 10.1155/2022/4640788

**Published:** 2022-03-18

**Authors:** Muhtasham Sifaat, Pinak Patel, Razan Sheikh, Dawood Ghaffar, Hitesh Vaishnav, Ludmila Nahar, Sonia Rupani, Syed Quadri

**Affiliations:** ^1^DeBusk College of Osteopathic Medicine, Lincoln Memorial University, 9737 Cogdill Rd, Knoxville, TN 37932, USA; ^2^Department of Medicine, School of Medicine/John D. Bower School of Population Health, University of Mississippi Medical Center, Jackson, MS 39216, USA; ^3^School of Medical Sciences, Lincoln Memorial University, 9737 Cogdill Rd, Knoxville, TN 37932, USA

## Abstract

Coronavirus disease 2019 (COVID-19) is an illness caused by a novel coronavirus called severe acute respiratory syndrome coronavirus 2 (SARS-CoV-2). Mutations in the genetic coding and the variations in the spike proteins are critical for the virus's mechanism of facilitating fusion with the human host, making the disease more severe. Recent research indicates that comorbidities including diabetes, hypertension, renal disease, heart failure, and atherosclerosis play a significant role in the severity and high mortality rates of (COVID-19), suggesting that perhaps the metabolic syndrome and its components are associated with COVID-19 morbidity. Primarily, angiotensin-converting enzyme 2 (ACE2) receptor is identified as the entrance receptor of SARS-CoV-2. Increased ACE2 expression, endothelial dysfunction plays a vital role in the progression and severity of complications developed due to COVID-19. In this review, we will discuss the association and management of cardiorenal disease and COVID-19.

## 1. Introduction

The novel coronavirus disease 2019 (COVID-19) is defined as an illness caused by severe acute respiratory syndrome coronavirus 2 (SARS-CoV-2), which has had a terrible effect on the world's demography, resulting in the deaths of over 5.89 million people globally [1]. After the first case of this primarily respiratory viral illness was reported in late December 2019 in Wuhan, Hubei Province, China, SARS-CoV-2 quickly spread around the world, prompting the World Health Organization (WHO) to proclaim it a pandemic [2]. Despite significant progress in clinical research leading to a better understanding of SARS-CoV-2 and the management of COVID-19, limiting the virus and its variants' continued spread has become an issue of increasing concern, as SARS-CoV-2 continues to wreak havoc around the world, with many countries experiencing a second or third wave of outbreaks especially attributed to the emergence of mutant viral strains.

While adapting to their new human hosts, SARS-CoV-2 is susceptible to genetic evolution with the formation of mutations over time, resulting in mutant variations with different characteristics than their ancestral strains. Novel Pfizer-BioNTech COVID-19 vaccine was recently approved by the Food and Drug Administration (FDA) [3]. Other vaccines and treatment have become available under emergency use authorization [4] to mitigate the spread and burden of COVID-19. However, their effectiveness and long-term outcomes are still to be determined. The emergence of newer SARS-CoV-2 variants could also threaten to undermine the significant progress made so far in limiting the spread of this viral infection [5].

The binding of spike protein to the ACE2 receptor leads to activation and progression of the infection [6]. The spike proteins cleave at the S1/S2 site after binding to ACE2, and the following cleavage of the S2 site enables the spike protein to fuse to the host membrane via irreversible conformational changes [7]. ACE2 is mainly expressed in the heart, lungs, intestinal epithelium, vascular endothelium, and kidneys, indicating its pivotal role in multiorgan dysfunction seen with SARS-CoV-2 infection [8, 9]. According to a recent study, COVID-19 is increasingly being linked to a higher risk of morbidity and mortality from cardiovascular disease (CVD) [10]. In this review, we will discuss the correlation between cardiorenal disease and COVID-19.

## 2. Methodology

The authors searched PubMed and Google Scholar for articles using the keywords “COVID-19”, “SARS-CoV-2”, “renin angiotensin aldosterone system”, “heart”, “cardiac”, “cardiovascular”, “heart failure”, “hypertension”, “atherosclerosis”, “kidney”, “renal dysfunction”, and “angiotion-converting enzyme inhibitors”. Case reports, systematic reviews and meta-analyses, clinical guidelines, and narrative reviews on COVID-19 and cardiovascular and renal effects and consequences were included. The research was limited to studies that were written in English.

## 3. COVID-19 in Cardiovascular Patients

Cardiovascular disease is the most prevalent comorbidity associated with COVID-19 [11]. In patients with CVD, COVID-19 can infect cardiac cells and cause significant heart damage through multiple mechanisms including an increase in renin-angiotensin aldosterone [12] system (RAAS) and proinflammatory mechanisms [13] leading to endothelial dysfunction, having a severe impact on their health [14, 15]. RAAS plays a vital role in blood pressure regulation and fluid balance. A hyperactive RAAS significantly increases angiotensin II which increases blood pressure and promotes sodium retention, vasoconstriction, oxidative stress resulting in ROS-mediated second messenger signaling, endothelial dysfunction, and organ damage [16, 17]. RAAS is also mediated by angiotensins 1-7 which counteracts the effects of angiotensin II [12, 18]. Angiotensin-converting enzyme 2 (ACE2) degrades angiotensin I (ang I) to inactive angiotensins 1-9 and angiotensin II (ang II) to angiotensins 1-7. Ang 1-7 exerts antioxidant, antifibrotic, vasodilators, and anti-inflammatory through Mas receptor [19] and counterbalances ACE-/ang II-mediated effects. ACE2 has been shown to mediate the viral entry of the severe acute respiratory syndrome coronavirus 2 (SARS-CoV-2) responsible for COVID-19 [6, 20]. This potentially leads to the reduction of ACE2, and the effects of ang II are augmented in the absence of RAAS-counterregulating mechanisms.

### 3.1. Hypertension

Data from China's National Health Commission suggests that 35% of COVID-19 patients also had hypertension [21]. According to a study, comparing 126 COVID-19 patients with a preexisting hypertension to 125 age and gender-matched COVID-19 patients without hypertension concluded that hypertensive patients had 21.3% more severe SARS-CoV-2 infection and a higher mortality rate (10.3% vs 6.4%) [13]. A group of 191 COVID-19 patients from Wuhan, China, were studied, and hypertension was present in 48% of nonsurvivors [22]. In a group of 138 hospitalized patients, hypertension was present in 58% of patients requiring ICU admission [21] establishing that hypertension is a major risk factor with worse clinical outcomes. The exact mechanism underlying these interactions is unknown. However, in patients with SARS-CoV-2 infection, hypertension could potentially exacerbate the inflammatory profile indicating that hypertension raises the chance of a more severe illness as these patients have greater levels of inflammatory biomarkers including tumor necrosis-*α* (TNF-*α*) and interleukin-6 (IL-6) contributing to endothelial dysfunction and oxidative stress confirming a possible pathophysiological link between hypertension and COVID-19 [12, 23].

### 3.2. Heart Failure

In COVID-19 patients, heart failure is another major cause of mortality. The mechanism of COVID-19's cardiac involvement is being investigated. Different myocardial mechanisms contribute to heart failure in COVID-19 patients, including direct myocardial involvement mediated by viral action [24]. Involvement of ACE2 was reported in a mouse model, SARS-CoV-2 infection resulted in an ACE2-dependent myocardial infection [25]. Another mechanism could be a potential cytokine storm, mediated by an unbalanced response across T helper cells [22], oxygen supply-demand imbalance, and hypoxia-induced increased intracellular calcium leading to cardiac myocyte death [21]. Proinflammatory biomarkers (D-dimer, ferritin, interleukin-6, and lactate dehydrogenase) and respiratory distress have been reported to exacerbate preexisting left ventricle (LV) failure or contribute to the development of new-onset cardiomyopathy [26]. Myocarditis, stress cardiomyopathy, or myocardial ischemia can all cause new-onset LV dysfunction. In a research by Zhou et al., nonsurvivors from COVID-19 had a higher rate of heart failure than survivors [22, 27]. Right heart failure is most likely caused by increased pulmonary artery pressure due to pulmonary dysfunctions. Exacerbation of heart failure with preserved ejection fraction can occur in the early stages of the disease due to vigorous fluid resuscitation attempts, and acute systolic heart failure leading to cardiogenic shock can occur in the advanced stages of the disease as cytokine levels rise [26, 27]. According to Guo et al., an increase in serum troponin in patients with or without previous cardiovascular disease was linked to an increase in plasmatic NT-proBNP levels, which increased mortality and was described as a poor prognostic marker [28]. As a result, heart disease is a significant risk factor for COVID-19 individuals.

### 3.3. Atherosclerosis

Inflammation has become a significant concern in COVID-19 patients encouraging the progression of atherosclerosis, leading to cardiovascular problems [29]. The primary issues associated with immunopathology in individuals with COVID-19 are dysregulation impacting T lymphocytes and an uncontrolled inflammatory process [30] supported by the fact that the blood samples from COVID-19 patients have reduced T lymphocyte, helper, and regulatory lines [31] resulting in uncontrolled inflammation. Another possible mechanism responsible for the inflammation in COVID-19 patients is cytokine storm [32, 33]. Recently, Huang et al. reported that interleukins (IL-1*β*, IL-7, IL-8, IL-9, and IL-10), fibroblast growth factor (FGF), tumor necrosis factor-*α* (TNF-*α*), granulocyte colony-stimulating factor (G-CSF), interferon (IFN), monocyte chemoattractant protein (MCP1), and macrophage inflammatory protein 1-*α* (MIP1-*α*) were significantly upregulated in the COVID-19 patients [34] An increase in von Willebrand factor antigen, von Willebrand factor activity, and factor VIII levels also contributes to the atherosclerotic plaque. Decreased antiaggregatory prostacyclin production and enhanced proaggregatory thromboxane synthesis from activated platelets can tilt the homeostatic situation towards a prothrombotic and proinflammatory phenotype in endothelial cells [35–37].

## 4. COVID-19 in Renal Disease Patients

Kidney disease is defined as an impaired glomerular filtration rate (GFR) or structural abnormalities as measured by albuminuria or abnormal urine analysis for 3 or more months [38]. The glomerulus is the filtration unit of the kidney, and an increase permeability of albumin is an early sign of renal dysfunction and kidney disease. It is a well-known fact that hypertension [16] and diabetes [39] promote kidney injury and contribute to renal failure particularly in elderly patients [40] and could predispose these individuals to COVID-19 infection. Recent systemic reviews and meta-analysis of the COVID-19 patients revealed that the prevalence of chronic kidney disease (CKD) was 9.1% worldwide [40–42] and about 15% in the United States of America [43]. A meta-analysis from Fisher et.al. showed that about 20% of patients with kidney disease and comorbidity had a 3-fold higher risk of contracting viral infection than those without CKD. Furthermore, patients with acute kidney injury (AKI) who tested positive for COVID-19 had a higher risk of CKD [44] than those who did not have AKI [45], recognizing CKD plays a key role in the severity of COVID-19.

The kidney is a likely target for COVID-19 due to its high number of cellular ACE2 receptors. These receptors are mainly localized in the glomerulus, mesangial cells, podocytes, and distal nephron [46–48] As discussed earlier, ACE2 is counterregulatory to local and systemic RAAS; therefore, decrease in ACE2 increases ang II accumulation and promotes albuminuria [46] suggesting its role in glomerular permeability. Additionally, protein efflux could be due to upregulation of angiotensin II receptor type 1 (AT1R) and prorenin receptor- (PRR-) activating proinflammatory pathways via the Wnt-catenin-snail pathway causing podocyte injury and actin rearrangement [49]. In hypertension and renal disease, it is shown that ACE2 expression is decreased promoting glomerular hypertrophy and proteinuria [50]. In diabetic kidney disease, ACE2 expression was found to be significantly decreased resulting in the formation of reactive oxygen species, kidney fibrosis, collagen deposition, mesangial matrix expansion, and podocyte loss [51]. This evidence indicates that disruption of RAAS homeostasis via downregulation of ACE2 and increase in ACE/ang II/AT1R activity impairs renal function and contributes to the COVID-19 infection, hospitalization, and mortality.

## 5. Management of Cardiorenal Complications in COVID-19 Patients

COVID-19 patients have higher levels of inflammatory cytokines. Both intensive care unit (ICU) patients and non-ICU patients with SARS-CoV-2 infection reported significantly higher plasma concentrations of interleukins and other cytokines than healthy subjects [34]. In cardiorenal diseases, ang II is responsible for cell proliferation, fibrosis, and hypertrophy by acting as a vasoconstrictor, proinflammatory, and prooxidative hormone which increases salt and water retention [16, 52] leading to end-organ dysfunction. RAAS blockers such as angiotensin-converting enzyme inhibitors (ACEI) and angiotensin receptor blockers (ARBs) are the most commonly used drugs in patients with hypertension, heart failure, and chronic kidney disease as they provide end-organ protection and increase ACE2 expression responsible for vasodilatory effects [53, 54]. Due to concerns that elevated ACE2 expression facilitates SARS-CoV-2 infection, the theory of a negative impact of RAAS inhibitors gained popularity early in the pandemic [55]. However, recent studies have challenged those theories and showed that the RAAS inhibitors can be safely used in COVID-19 patients with cardiorenal comorbidities [56].

Review of the literature and a recent meta-analysis of 52 studies with more than 10000 patients contributes to a growing body of evidence indicating that the ACE-I/ARBs do not harm COVID-19 patients and in fact provide protective benefits by controlling the blood pressure which lowers the risk of mortality [57]. Another study comprising of more than 17000 COVID-19 patients reported that the ACE-I/ARBs decrease the need of ICU admission, mechanical ventilation, and progression of critical pneumonia with multiple mixed comorbidities [58]. Taken together, these studies indicates that the RAAS antagonist plays a protective role in COVID-19 across all patient groups. Patients who take other blood pressure medications have shown poorer clinical outcomes and are more likely to have significant adverse effects than those who take ACE-I/ARBs. As a result, the international scientific community including American Heart Association recommends that COVID-19 individuals with metabolic syndrome receive ACE-I/ARBs unless contraindicated.

ACE-I/ARBs mediate its cardiorenal protective effects by inhibiting fibrosis and inflammation. Research have proved that ACE-I/ARBs decrease proinflammatory markers including IL-6, IL-1*β*, MCP-1, NF-*κ*B, TNF-*α*, C-reactive protein, intracellular adhesion molecule-1, and vascular adhesion molecule-1 [59, 60]. These effects could in part due to unopposed activation of angiotensin type 2 receptor (AT2R) and increase in expression of inhibitory kappa B [61].

There are certain limitations to this review, because research with higher levels of evidence, such as randomized clinical trials, were unavailable.

## 6. Conclusion

COVID-19 patients frequently have cardiorenal comorbidities, putting them at a higher risk of morbidity and mortality. The use of a clinically approved RAAS antagonist should be continued, according to the available data. Although this study emphasizes the link between RAAS inhibitors and mortality in COVID-19 patients, further randomized trials are required to demonstrate correlation.
